# The Feasibility of the “Omega Kid” Study Protocol: A Double-Blind, Randomised, Placebo-Controlled Trial Investigating the Effect of Omega-3 Supplementation on Self-Regulation in Preschool-Aged Children

**DOI:** 10.3390/nu13010213

**Published:** 2021-01-13

**Authors:** Lauren A. Roach, Mitchell K. Byrne, Steven J. Howard, Stuart J. Johnstone, Marijka Batterham, Ian M. R. Wright, Anthony D. Okely, Renate H. M. de Groot, Inge S. M. van der Wurff, Alison Jones, Barbara J. Meyer

**Affiliations:** 1 School of Medicine, Faculty of Science, Medicine and Health, University of Wollongong, Wollongong, NSW 2522, Australia; lroach@uow.edu.au; 2 School of Psychology, Faculty of the Arts, Social Sciences and Humanities, University of Wollongong, Wollongong, NSW 2522, Australia; stevenh@uow.edu.au; 3 Illawarra Health and Medical Research Institute, University of Wollongong, Wollongong, NSW 2522, Australia; ian.wright@jcu.edu.au (I.M.R.W.); bmeyer@uow.edu.au (B.J.M.); 4 Early Start, School of Education, Faculty of the Arts, Social Sciences and Humanities, University of Wollongong, Wollongong, NSW 2522, Australia; tokely@uow.edu.au; 5 Brain & Behaviour Research Institute, School of Psychology, Faculty of the Arts, Social Sciences and Humanities, University of Wollongong, Wollongong, NSW 2522, Australia; sjohnsto@uow.edu.au; 6 Statistical Consulting Centre, School of Mathematics and Applied Statistics, Faculty of Engineering and Information Sciences, University of Wollongong, Wollongong, NSW 2522, Australia; marijka@uow.edu.au; 7 College of Medicine and Dentistry, James Cook University, Cairns, QLD 4870, Australia; 8 Conditions for Lifelong Learning, Faculty of Educational Sciences, Open University of the Netherlands, 6419 Heerlen, The Netherlands; renate.degroot@ou.nl (R.H.M.d.G.); inge.vanderwurff@ou.nl (I.S.M.v.d.W.); 9 Faculty of Science, Medicine and Health, University of Wollongong, Wollongong, NSW 2522, Australia; alisonj@uow.edu.au; 10 School of Medicine, Molecular Horizons, Faculty of Science, Medicine and Health, University of Wollongong, Wollongong, NSW 2522, Australia

**Keywords:** n-3 LCPUFAs, self-regulation, preschool-aged children, feasibility, executive function

## Abstract

Self-regulation, the regulation of behaviour in early childhood, impacts children’s success at school and is a predictor of health, wealth, and criminal outcomes in adulthood. Self-regulation may be optimised by dietary supplementation of omega-3 long-chain polyunsaturated fatty acids (n-3 LCPUFAs). The aim of the “Omega Kid” study is to investigate the feasibility of a protocol to investigate whether n-3 LCPUFA supplementation enhances self-regulation in preschool-aged children. The protocol assessed involved a double-blind, randomised, placebo-controlled trial of 12 weeks duration, with an intervention of 1.6 g of eicosapentaenoic acid (EPA) and docosahexaenoic acid (DHA) per day (0.3 g EPA and 1.3 g DHA) in a microencapsulated powder compared to placebo. Children (n = 78; 40 boys and 38 girls) aged 3–5 years old were recruited and randomly allocated to the treatment (n = 39) or placebo group (n = 39). The HS–Omega-3 Index^®^ served as a manipulation check on the delivery of either active (n-3 LCPUFAs) or placebo powders. Fifty-eight children (76%) completed the intervention (28–30 per group). Compliance to the study protocol was high, with 92% of children providing a finger-prick blood sample at baseline and high reported-adherence to the study intervention (88%). Results indicate that the protocol is feasible and may be employed in an adequately powered clinical trial to test the hypothesis that n-3 LCPUFA supplementation will improve the self-regulation of preschool-aged children.

## 1. Introduction

Self-regulation is the ability to control one’s emotions and cognition to execute planned behaviours, problem-solve and control impulses [1]. Self-regulation comprises of three inter-related domains: cognitive self-regulation (including problem-solving, attention, decision-making and cognitive flexibility), emotional self-regulation (including controlling strong emotions, empathy for others and self-calming), and behavioural self-regulation (including adhering to rules, impulse control and goal-orientated behaviour) [1]. Disruptions in the development of self-regulation are symptomatic of a number of disorders, including attention deficit hyperactivity disorder (ADHD) [2] and autism spectrum disorder (ASD) [3]. In early childhood, poor self-regulation has a number of consequences for early primary school performance in language and literacy skills, as children with poorer self-regulation in preschool are more likely to have poorer academic performance [4] and display more problematic externalising behaviour [5]. 

However, the implications of poor self-regulation abilities in early childhood appear to have even more impact, with an array of apparent long-term consequences. A recent meta-analysis of 150 studies shows that self-regulation capacity in preschool is predictive of social competency, school engagement and academic performance outcomes in early schooling, academic achievement in later school years and negatively related to a number of poor health behaviours in adulthood [6]. A longitudinal study by Moffit et al. [7] found that self-regulation from as early as age 3 was predictive of adolescent and adult outcomes in health (cardiovascular, respiratory, dental, sexual health and inflammatory status), wealth (income, socioeconomic status, financial planning and financial struggles) and criminal convictions by age 32 [7]. Comparatively, children who became more self-controlled from childhood to young adulthood (for whatever reason, as the study of Moffit et al. was not an intervention study) had better outcomes at 32 years of age compared to children who did not improve their rank order of self-control [7]. This suggests that (1) self-regulation in childhood can be improved, and (2) improvements can result in better long-term outcomes, even into adulthood. 

Executive functions are a core component of cognitive self-regulation [8] and are comprised of three factors: shifting between tasks, updating working memory, and inhibition of dominant responses [9]. Given that executive functions are also malleable, they have become a notable target to improve self-regulatory abilities in children [8]. A number of interventions have been carried out in children and adolescents (aged 2 to 17 years) to improve self-regulation and executive function. These interventions include curriculum-based exercise (e.g., yoga, martial arts, wellness) and family and social skills or personal skills interventions [10]. The majority of these interventions have shown improvements in self-regulation and also beneficial effects in distal outcomes such as academic achievement, substance abuse, conduct disorders, social skills, depression, behaviour problems and school suspensions [10]. However, interventions such as these are resource-intensive and difficult to disseminate widely and therefore not feasible for large cohorts. Interventions which are feasible and easy to administer to preschool-aged children are needed, considering that in the absence of an intervention, 20% of children with poor self-regulation do not improve in the final year before school, with impacts upon school readiness, and that by age 7, a concerning number (18–35%) still have self-regulation levels typical of 4-year-olds [11]. 

Given the importance of developing an age-appropriate self-regulatory capacity before commencing school and the pragmatic limitations of extant intervention strategies, a need exists to investigate more broadly disseminable interventions. Few studies have employed or assessed the feasibility of nutritional interventions by means of diet or supplements as a means to enhance self-regulation. This is noteworthy as it is well known that certain nutrients such as omega-3 long-chain polyunsaturated fatty acids (n-3 LCPUFAs) are required for neurological development [12], and are vital throughout the lifespan, including in early childhood [13,14]. Of the n-3 LCPUFAs, docosahexaenoic acid (DHA) is the prominent fatty acid in the brain, which accumulates in areas of the brain responsible for working memory, such as the frontal cortex [15]. N-3 LCPUFAs are part of a healthy balanced diet and are primarily found in seafood sources such as fish [16]. In Australia, consumption of foods rich in n-3 LCPUFAs is relatively low, as 80% of children do not regularly consume fish/seafood [17]. 

From a physiological health perspective, both DHA and eicosapentaenoic acid (EPA) are necessary for optimal brain development and function. Previous studies that have investigated the effects of n-3 LCPUFAs in typically developing preschoolers (3–6 years) generally report benefits to vocabulary [18], cognitive performance in regards to memory and spatial sense [19], and higher scores on an intelligence scale [20]. These outcomes result from either n-3 LCPUFA supplementation studies [18,19] or fatty fish consumption studies [20] in this age group, and range from 8 to 16 weeks in duration. The study of Ryan and Nelson [18] reported high adherence to the treatment, suggesting the n-3 LCPUFA supplementation is an acceptable and feasible intervention in this age group. However, with such a paucity of studies of this nature conducted on preschool-aged children, further work is needed to confirm the acceptability and feasibility of such interventions and the related study procedures by participating families. In children of primary school age (5–13 years), there is a range of evidence supporting the use of n-3 LCPUFAs for ADHD symptoms, in children with diagnosed ADHD and in groups with no diagnosed ADHD. Intervention trials reported benefits of n-3 LCPUFA to working memory [21] and disruptive behaviour and attention [22] in children with ADHD. Furthermore, there were benefits to ADHD symptoms and other symptoms such as cognition, anxious and shy behaviour, hyperactivity/impulsivity [23], reading and Conners’ behavioural scores [24] observed in children with no ADHD diagnosis, suggesting that a range of self-regulatory abilities can benefit. Even in mainstream schoolchildren, n-3 LCPUFAs have shown improvements to reading ability, visual perception and impulsivity [25,26].

However, implementing such trials in the early childhood group presents unique challenges such as children will refuse to complete study assessments or consume supplements; therefore, any such protocol must be thoroughly tested prior to wider dissemination. Given the paucity of evidence and potential challenges of intervening in the 3–5 age group, the primary aim of the Omega Kid Study is to investigate the feasibility of conducting a 12-week omega-3 intervention (1.6 g of EPA and DHA), including a finger-prick omega-3 assessment, in preschool-aged children. The study addresses the feasibility of measurement of the effects of n-3 LCPUFA supplementation on self-regulation, executive function, electroencephalographic (EEG) measures, and ADHD symptoms. Furthermore, this study assesses the acceptability of the study procedures, such as the finger-prick assessment and intervention by the participating families. 

### Hypothesis

The study protocol and implementation of the intervention of n-3 LCPUFA supplementation and assessment of outcomes will be feasible for use in preschool-aged children.

## 2. Materials and Methods 

### 2.1. Study Design

A randomised, double-blind, placebo-controlled trial with a parallel design was executed to determine the feasibility of an n-3 LCPUFA supplementation intervention for self-regulation in preschool-aged children compared to placebo supplementation. The intervention period was 12 weeks, and assessments occurred at baseline and postintervention. 

### 2.2. Ethics Approval and Clinical Trial Registration

The study was conducted in accordance with the Declaration of Helsinki. This study was approved by the University of Wollongong Human Research Ethics Committee (Approval Number: 2019/171) and registered with the Australian New Zealand Clinical Trial Registry (Registration Number: 12619000731190). This manuscript adheres to the CONSORT statement. 

### 2.3. Participants, Inclusion and Exclusion Criteria 

Children were eligible for this study if they were aged between 3 and 5 years. Children already consuming fish oil could be included in the study provided the total daily dose of EPA and DHA (including the study dose) did not exceed 3 g/day [27]. Children were excluded if they were allergic to the allergens in the study treatments (fish, milk and soybean), had blood-clotting disorders, were on blood-thinning medication, had an upcoming surgical procedure, or had known developmental delay (other than ADHD).

### 2.4. Recruitment

Families were recruited through long-day care centres, playgroups and University networks in an area of approximately 100 km radius from the University of Wollongong. After approval from the daycare centre director, families with children aged 3–5 years were approached and invited to participate in the study. An animated video was used to assist with recruitment, which was placed on the child care centres’ newsletter or communication platform with parents (https://youtu.be/jVeba3F5Rpw). Families that completed the baseline assessments were given passes to a local children’s museum (Early Start Discovery Space https://www.earlystartdiscoveryspace.edu.au/) for up to 4 people or a gift voucher to the children’s museum gift shop of equal value. Families that completed the study were additionally given a year’s membership to the children’s museum (for up to 4 people) or a gift voucher to the children’s museum gift shop of equal value. 

### 2.5. Randomisation and Blinding of Study Intervention

Children were randomly assigned to one of two groups (Groups A and B) without stratification. Randomisation was conducted using Stata statistical software (Version 16, College Station, TX, USA) by a statistician in order to randomise the study codes against either treatment code in a 1:1 allocation ratio. Randomisation and group assignment was fully concealed from the researchers, children and parent or caregivers for the entire study and will continue throughout further data analysis (analysis of treatment effect on behavioural outcome measures to be published following data analysis). 

Children were randomly allocated to the n-3 LCPUFA or placebo group for the 12 weeks of daily powder consumption. The powder of the n-3 LCPUFA group consisted of microencapsulated tuna oil powder (Driphorm^®^ HiDHA^®^ 50), manufactured and supplied in kind by Nu-Mega Ingredients (Altona North, Victoria, Australia), containing 11.6% *w*/*w* DHA and 2.7% *w*/*w* EPA, which enriched the n-3 LCPUFA powder to deliver 1.3 g of DHA and 0.3 g of EPA per day. The placebo powders contained an equivalent amount in weight of high oleic acid sunflower oil instead of the n-3 LCPUFA powder. 

Both powders were delivered in a base vanilla-flavoured powder in individual sachets labelled A or B to ensure the treatments were sufficiently blinded. Parents and caregivers were advised to deliver the supplements daily to the children with or immediately after the evening meal by mixing into either warm milk or vanilla yoghurt to increase the bioavailability of the supplements. Consumption was recorded on a self-check calendar provided to parents/caregivers. 

### 2.6. Data Collection

Participants (children and parents/caregivers) attended two clinic visits between September and December 2019 at the Child and Adolescent Psychology Clinic at the University of Wollongong: one at baseline and one after 12 weeks of intervention. Table 1 is a summary of the outcome measures and testing methods. Clinic visits took approximately 1 h each. During each of these visits, participating children completed practical assessments whilst the parent/caregiver completed behavioural questionnaires about their child. After the assessments at both baseline and postintervention, the child provided a finger-prick blood sample. The schedule of the study is provided in Table 2. 

### 2.7. Blood Collection

Capillary blood samples were collected at baseline and postintervention (after 12 weeks) to determine the HS–Omega-3 Index^®^ [33]. Samples were nonfasted; however, parents/caregivers were advised to not feed their child foods high in n-3 LCPUFA, namely, fish but also eggs and meat, on the morning of the sampling. Samples were collected using a sterile, single-use lancing device (Accu-Chek^®^ Safe-T-Pro Plus, Roche Diabetes Care, North Ryde, New South Wales, Australia) to collect a whole-blood capillary finger-prick sample on a filter paper embedded with a stabiliser provided by Omegametrix(Martinsried, Bavaria, Germany). These samples were stored at −80 °C and then bulk-shipped on dry ice to Omegametrix, Martinsried, Germany, to be analysed for fatty acid composition using Omegametrix standard methodology [34]. The HS–Omega-3 index^®^ served as a manipulation check of the intervention. 

### 2.8. Feasibility of Finger-Prick Blood Sample

The feasibility of finger-prick blood collection was measured by the number of children who provided a sample at both baseline and postintervention. The acceptability of finger-prick capillary blood collection by both parent/caregivers and participating children was also assessed. Parents/caregivers had to indicate their level of agreement to the following single 5-point Likert Scale question: “*Now that the finger-prick blood sample has been collected*/*attempted, I believe that this procedure is acceptable in a research setting for preschool-aged children”.* Responses ranged from Completely Disagree, Somewhat Disagree, Not Sure, Somewhat Agree or Completely Agree. Children were assessed using a pictorial “smiley face” 3-option Likert Scale question, with either a “happy” face, a “neutral” face or a “sad” face. 

### 2.9. Compliance to Study Protocol

Parents or caregivers were provided with a self-check calendar to tick off when the sachets were consumed or if a dose was refused or missed. These were returned at the end of the study for researchers to calculate the total number of doses consumed and to provide an indirect measure of compliance, second to the HS–Omega-3 Index^®^. The self-check calendar was compared with returned (unused) sachets at the conclusion of the intervention as a double-check on compliance. 

Families were encouraged to contact researchers to report any adverse events. In addition, researchers contacted the families throughout the study via email, which gave families an opportunity to discuss any problems or report any adverse events.

### 2.10. Acceptability of Study Intervention

An end-of-trial questionnaire was completed by parents/caregivers at the final clinic visit. This questionnaire was used to assess the parent/caregiver experience with the study and appraise the perceived acceptability and feasibility of the study protocol by parent/caregivers, providing qualitative feedback to the researchers. Questions included, “Did your child like the supplements?” and “Did you find the sachets easy to administer to your child?”.

### 2.11. Completion Rate of Children and Parent/Caregiver Tasks

To assess the feasibility of the study protocol, the completion rate for all measures completed by both children and parents/caregivers listed below were recorded and expressed as a percentage of participants who completed the individual tasks. 

#### 2.11.1. Self-Regulation

Self-regulation was assessed by the Head, Toes, Knees, Shoulders (HTKS) task [28] completed by the children and the Child Self-Regulation and Behaviour Questionnaire (CSBQ) [29] completed by the child’s parent/caregiver. The HTKS task asks children to remember correspondences between body parts (e.g., head and knees), and enact the opposite action to what was instructed (e.g., touch their knees when told “touch your head”) [28]. The HTKS task was conducted by a research assistant who was trained in this assessment and had experience administering the task. 

The CSBQ is a validated 34-item adult-report questionnaire of children’s self-regulation, with each item having a scale response of 1 to 5 (from “not true” to “certainly true”). 

#### 2.11.2. Executive Function

Executive function was assessed by the validated Early Years Toolbox iPad tasks “Go/No-Go” and “Mr Ant” [29], which were completed by the participating children. The “Go/No-Go task” is a measure of inhibition (a core executive function). On an iPad, children are presented with fish and sharks swimming across the screen and instructed to tap the iPad screen when they see a fish (“catch the fish”) and refrain from responding when a shark appears (“avoid the sharks”) [29]. 

The “Mr Ant” task is a measure of working memory (another core executive function). During this task, children are asked to remember the location of coloured dots on a cartoon ant. When the coloured dots disappear, children are asked to indicate the location of these dots by tapping the screen where they believe the dots were initially [29]. 

Executive function was also assessed by parents/caregivers using the Behaviour Rating Inventory of Executive Functioning—Preschool Version (BRIEF-P) [30], a 73-item questionnaire. 

#### 2.11.3. EEG Measures

An EEG recording provides a neurobiological measure of cognitive functioning that can be compared to observed behaviour. For this study, EEG was measured using a wireless, single-channel, dry-sensor, portable measurement device (the Neurosky^®^ MindWave Mobile 2^TM^ headset, San Jose, CA, USA). The EEG recording took place during both resting (eyes-open and eyes-closed for 30–60 s each) and active (during the iPad task “Go/No-Go”) states. The EEG data were recorded to a PC for processing and quantification, deriving estimates of power in the delta, theta, alpha, and beta EEG bands. Data recorded from this device have been shown to be valid and reliable and show sensitivity to psychological states [35,36,37].

#### 2.11.4. ADHD

Symptoms of ADHD were assessed using the Conners’ Teacher Rating Scale-15 (CTRS-15) [31]. Given that ADHD is characterised by impairments in self-regulation, the CTRS-15 will be used as an indirect measure of self-regulation. This questionnaire was designed for preschool-aged populations and was completed by a parent/caregiver of the participating child.

#### 2.11.5. N-3 LCPUFA Dietary Intake

An electronic Polyunsaturated Fatty Acid Food Frequency Questionnaire (PUFA FFQ) [32] was completed by the participating child’s parent/caregiver regarding the child’s usual diet at baseline. The PUFA FFQ consists of 38 questions regarding usual dietary habits over the past three months. The questionnaire is automated to calculate PUFA intake and provide an estimate of n-3 LCPUFA intake.

### 2.12. Statistical Analysis

All participants’ personal details (names and contact details) were stored in a separate Excel file. All hard copies and electronic data were deidentified using a study code, accessible only by the research team. Hard copies of data were securely stored by members of the research team for the duration of the study, to be kept for 15 years before disposal in confidential waste bins for commercial shredding. Hard copies of questionnaires were entered into Excel, and, for all paper-based questionnaires, at least 10% were randomly selected and checked for data entry accuracy.

Data were analysed by IBM^®^ SPSS^®^ Statistics (Version 25, IBM Corp, Armonk, NY, USA), with relevant assumptions tested. Data are presented as mean (standard deviation) for normally distributed variables, median (25th, 75th percentiles) for non-normally distributed variables, and as proportions and percentages for categorical data.

To determine the difference between groups (difference in demographics, difference between those providing a finger-prick sample and difference in likeability of supplements), *t*-tests, Mann–Whitney tests or chi-squared tests were used. Significance was determined using an alpha level of 0.05. All tests were 2-tailed.

## 3. Results

### 3.1. Recruitment and Retention

**Figure 1:**
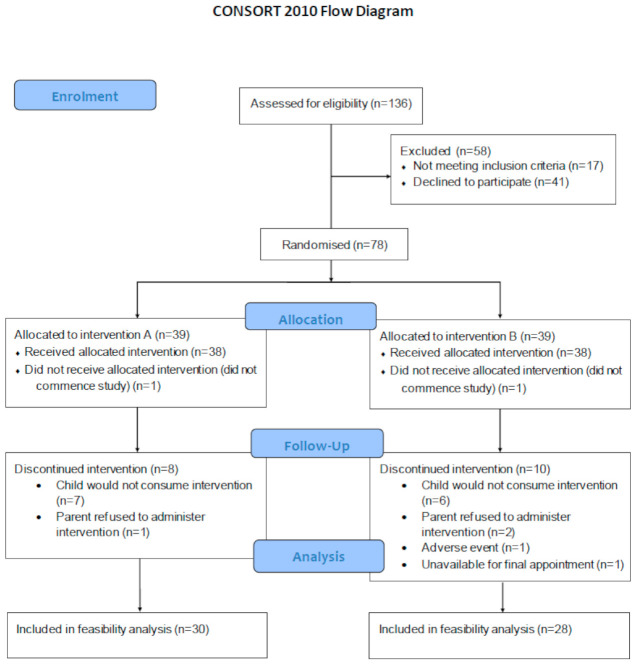
CONSORT diagram of recruitment, randomisation, allocation and follow-up of the children included in the feasibility analysis.

A total of 78 children were eligible to participate and were randomised into the omega-3 or placebo group. Thirty-nine children were allocated to each intervention group; however, 1 child/family withdrew from the study in each group, leaving 38 per group to commence (Figure 1). Gender distribution between groups was identical, with 20 boys and 18 girls in each group. There was no significant difference in age between the two groups (*p* = 0.175), with the median and interquartile range of 4.0 (3.5–4.8) and 4.4 (3.6–5.1) years and months for Groups A and B, respectively.

At the end of the intervention, the rate of retention for Group A was 79%, with 74% for Group B. Thus, the dropout rate was 24%, and, of the children who remained in the study, 98% provided data for analyses. Three adverse events (gastrointestinal issues) were reported from two participants in Group A and one participant in Group B. Of these, one participant in Group B withdrew while the other two continued in the study.

### 3.2. Enrolment and Randomisation to Intervention

See Figure 1.

### 3.3. Blood Collection

At the baseline clinic visit, 70 of the 76 children provided a finger-prick blood sample (92%). There was no difference in age or gender between those who agreed to provide a sample and those that did not, as determined by the Mann–Whitney test and Pearson’s chi-square, respectively (*p* = 0.330 (age) and *p* = 0.473 (gender)). At the final clinic visit, 50 children out of 58 (86%) provided a finger-prick blood sample. Once again, there was no difference in age (*p* = 0.322) or gender (*p* = 0.580) for those who agreed to provide a sample and those who did not. Of the 50 children who provided a sample at the final visit, 49 had also provided a sample at baseline. One child did not provide a sample at baseline but agreed to provide a sample at the final visit.

At baseline only, children were asked how they felt after the finger-prick collection, with 69 of 70 children providing a response. Of those, 79.7% gave a neutral (neither happy nor sad face) or positive (happy face) response and 20.3% gave a negative response (sad face) (Table 3). At baseline only, parents and caregivers were asked whether they believed a finger-prick sample was appropriate in a research setting for children of this age, with 94.3% giving a positive response (somewhat or completely agree) and 2.8% giving a negative response; 2.9% were not sure (Table 4).

### 3.4. Compliance to Treatment

Compliance to the study treatments was assessed by a calendar check sheet completed by parents/caregivers throughout the trial. Median levels of compliance were 86.9% for Group A and 89.3% for Group B, averaging 88% across groups. There was no significant difference in levels of compliance between groups, as determined by the Mann–Whitney test (*p* = 0.216). A check on returned sachets confirmed calendar responses with no significant difference detected between total dose calculated from calendar sheets or returned sachet count (determined from subset n = 46 who returned sachets; *p* = 0.746).

### 3.5. Acceptability of Study Intervention

Parents/caregivers were asked whether they believed their child liked, disliked or neither liked nor disliked the study supplement. A total of 59% (34 out of 58) of parents reported that their child liked the supplements, with no significant difference between groups (18 in Group A and 16 in Group B; *p* = 0.327). Conversely, 38% (22 out of 58) of parents reported that their child did not like the supplement, again with no significant difference between the groups (12 in Group A and 10 in Group B; *p* = 0.327). A further 2 children (3%) neither liked nor disliked the supplements (both were assigned to Group B).

Finally, parents/caregivers were asked if the study supplements in the individual sachets were easy to administer. Overall, 79.3%, 46 (23 from each group) out of 58 parents believed they were easy to administer. A further 17.2%, 10 (5 from each group) out of 58 parents, said they were not easy to administer. Lastly, 3.4% (2 out of 58; both children from Group A) of parents said neither yes nor no.

### 3.6. Collection of Outcome Measures

The children’s engagement in assessment tasks, completion of tasks, and adherence to instructions (thus yielding usable data) is detailed in Table 5. On average, 93% of the children completed assessments at baseline and, of those, 98.5% yielded interpretable data. At postintervention, 93% completed assessments and all data obtained (100%) were interpretable. Participation in the EEG tasks was poorer than other measures, with some children unwilling to wear the headset. Furthermore, in the case of the eyes-open and eyes-closed EEG measures, some children were unable to sit still for the required length of time needed for the recording (minimum 30 s).

Parental/caregiver engagement in the assessments was high. At baseline, all parents/caregivers completed the BRIEF and the CSBQ, while 75 out of 76 (99%) completed the Conners questionnaire. At postintervention, all behavioural questionnaires (BRIEF, CSBQ and Conners) were completed by parents. The PUFA FFQ was designed to be completed by parents at home after the baseline assessment; however, some parents completed the questionnaire closer to the final appointment. Sixty-seven of 76 (88%) parents completed the questionnaire; however, this included all the parents/caregivers of the 58 children who completed the intervention, suggesting that some families withdrew from the study before completing the questionnaire.

## 4. Discussion

This study evaluates the feasibility of a trial (“Omega Kid”) to investigate whether n-3 LCPUFA supplementation in 3- to 5-year-old children improves self-regulation and executive functioning. Feasibility issues assessed included supplement acceptability, outcome measure completion by both children and parents/caregivers, engagement in and acceptability of a manipulation check through blood collection, compliance, and retention in the study. The results obtained strongly support the feasibility of Omega Kid study protocol and support the viability of a larger study powered to assess whether n-3 LCPUFA supplementation can affect an improvement in young children’s self-regulation. Specifically, we observed a high retention rate of 76%; strong adherence with the manipulation check (completion of the finger-prick collection) of 92% at baseline and 86% at study completion; good child compliance, with assessments averaging 93% at both time points and nearly all data interpretable at study completion; near 100% compliance by parents/caregivers with their assessment tasks; 88% compliance with the administration of the supplement.

Parental acceptability of the study procedure is an important determinant of the success of a trial in a paediatric population [38]. Qualitatively, families reported that the study was acceptable, with 79% of the parents/caregivers reporting ease in the administration of the supplements and 59% suggesting that their children liked the supplements. Importantly, 94% of parents/caregivers confirmed that the finger-prick blood test was acceptable in research. Similarly, 80% of the children expressed neutral or positive responses to the finger-prick blood test.

A particular strength of the design of the Omega Kid study is the collection of a blood sample, which will enable both a confirmation of compliance with supplementation and a direct association between outcome measures and the child’s omega-3 index, as recommended by the International Society for the Study of Fatty Acids and Lipids’ (ISSFAL) Official Statement Number 6 [39]. The finger-prick blood sample collection was chosen in this group due to the less invasive and less painful nature of the procedure compared to venepuncture. Only one previous study of n-3 LCPUFA intervention in typically developing preschool-aged children also used finger-prick blood sampling; however, in that case, only just over half of the participants provided a sample at baseline and at the end of the intervention [18]. There was no further explanation as to why there was low completion of the finger-prick blood sampling and no examination of the children’s or parents’ response to the procedure. In our study, the success in obtaining blood samples and the positive responses of both parents and children to the experience were likely due to strategies implemented during the procedure. In every case, the parent/caregiver was present when the sample was obtained, and the process of collection included providing distraction and giving the child a reward following blood collection. These strategies have all been shown to be effective in reducing the perception of pain in young children. For example, a distraction during blood collection procedures, such as an animation, has been shown to reduce the pain response of young children during venepuncture [40,41], and, in some cases, a distraction was superior to anaesthetic cream for pain management [42]. In this study, children were distracted by a research assistant using toys and books. In previous research, the use of a reward after an immunisation procedure, such as a small toy, reduced the persisting perception of pain and facilitated coping in children after receiving the needle [43]. In this study, children were presented with a colouring book after their finger-prick blood collection. Lastly, it was important that parents remained with their child during the sampling, as this has been shown to alleviate some anxiety for similar procedures [44].

The form of the study supplement was a powder, which is easy to administer into other foods and can provide a high dose of n-3 LCPUFAs required for the study. This was the first study to use microencapsulated tuna oil in typically developing preschool-aged children for behavioural intervention and, therefore, assessing the acceptability of the intervention by participating families was required and important to inform future research. Other forms of supplements may present a choking hazard (capsules) or be difficult for families to store as they require refrigeration (oils and emulsions). The supplements were administered in a base vanilla powder that was flavoured to mask the fish taste of the omega-3 powder to ensure the participants were successfully blinded. Although the sachets were flavoured, a number of families reported their child did not like the taste of the sachets and they had trouble getting their child to consume them. The sachets were designed to be incorporated into vanilla yogurt or warm milk, but for families whose children would not consume the powders, other strategies were implemented, such as incorporating the powders into foods such as smoothies, porridge and custards. It is noted that this introduces some variability into the study as different foods may impact the bioavailability of the powders. While some families reported that their children did not like the sachets, the overall rate of compliance ranged from 86.9–89.3% per group, suggesting the palatability of the supplements was not a large determinant of overall consumption. Furthermore, the majority of parents indicated the supplements were easy to administer, which may have contributed to the overall high consumption. Minimal adverse events were reported. Driphorm^®^ HiDHA^®^ powders have been used previously in younger children at a lower dose and were also tolerated well [45].

None-the-less, the most common reason for participant withdrawal was that the children would not consume the sachets. A total of 38% of the children who completed the trial did not like the study supplements. However, when taking into account those who withdrew due to not liking the supplements, the total number of children enrolled in the study who did not like the supplements overall was 35 out of 76 or 46%. Future studies will need to investigate measures to enhance the palatability of the supplements.

While high completion rates were observed for the child assessments, the authors concede that the number of assessments was intense for children. Future studies should consider streamlining the assessments. The lowest adherence was for the EEG recording, with some children not wanting to wear the headset or, while wearing the headset, not sitting still and following the instructions for the eyes-open or eyes-closed recording sufficiently for a clean data recording. Depending on what the EEG data analysis yields, the intensity of the assessment protocol may be reduced by removing the EEG component, given the lower rate of usable data. This would also benefit sample size calculations by reducing the number of variables.

For parents, the data with the lowest completion rate was the PUFA FFQ. This may be because the PUFA FFQ was the only assessment completed at home in the participants’ own time. When participants are away from the clinic, they may be less motivated to complete the questionnaire. Therefore, future work should consider having parents complete all assessments during the clinic visits so that all data are complete.

## 5. Conclusions

In conclusion, the biometric protocol of the Omega Kid trial has shown to be feasible when implemented in a group of Australian 3- to 5-year-old children, including blood collection via finger-prick. Future work should take into account the palatability of the study supplements and the number of outcome measures requiring completion by participating children. To determine whether n-3 LCPUFA supplementation in this age group results in improvements in self-regulation, this protocol should be implemented to a larger sample in a multicentre, double-blind, randomised, placebo-controlled trial.

## Figures and Tables

**Table 1:** Summary of the study outcome measures and methods.

Variables	Methods	Who Completes the Task?
Self-regulation		
Head-Toes-Knees-Shoulders task	Child to follow instruction and enact opposite action to what was instructed (e.g., touch their knees when told to touch your head) [28]	Children during clinic visits
Child Self-Regulation and Behaviour questionnaire	34-item questionnaire [29]	Child’s parent or caregiver during clinic visits
Executive function		
Go/No-Go task	The “Go/No-Go task” is an iPad based measure of inhibition [29]	Children during clinic visits
“Mr Ant” task	The “Mr Ant” task is an iPad based measure of working memory [29]	Children during clinic visits
Behaviour Rating Inventory of Executive Functioning—Preschool Version (BRIEF-P)	The BRIEF-P is a 73-item questionnaire [30]	Child’s parent/caregiver during clinic visits
Other assessments		
EEG	A wireless, single-channel, dry-sensor, portable, measurement device (the Neurosky^®^ MindWave Mobile 2^TM^ headset)	A trained research assistant during clinic visits
ADHD questionnaire	Conners’ Teacher Rating Scale-15 (CTRS-15) [31]	Parent/caregiver during clinic visits
PUFA food frequency questionnaire	A link was provided and was completed online [32]	Parent/caregiver at home
Acceptability of finger-prick blood sampling	A single question Likert Scale A pictorial “smiley face” Likert Scale	Parent/caregiver during clinic visits Children during clinic visits
End-of-trial questionnaire	Open-ended questionnaire to assess experience with the study	Parent/caregiver during clinic visits
Manipulation check Whole blood fatty acids	Capillary blood using gas chromatography HS–Omega-3 Index^®^ [33]	Analysis conducted by Omegametrix

**Table 2:** Schedule of enrolment, intervention and assessments.

	Enrolment		Allocation	Postallocation	
TIMEPOINT	*−t*_2_	*−t*_1_	0	*t*_baseline_	*T*_12weeks_
ENROLMENT:					
Eligibility screen	X				
Informed consent	X				
Exclusions ^1^		X			
Allocation			X		
INTERVENTIONS:					
Omega-3				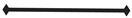	
Placebo				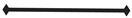	
ASSESSMENTS:					
Self-regulation					
Head, toes, knees, shoulder task				X	X
Child self-regulation and behavioural questionnaire				X	X
Additional outcomes Executive function					
Go/No-Go task				X	X
“Mr Ant” task				X	X
Behaviour rating inventory of executive functioning				X	X
EEG				X	X
Other assessments ADHD ^2^ questionnaire				X	X
PUFA ^3^ food frequency questionnaire				X	
Manipulation check—whole-blood fatty acids				X	X
Acceptability of finger-prick blood sampling				X	X
End-of-trial questionnaire					X
Adverse events				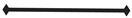	

^1^ Allergies to supplements, blood clotting disorders, blood-thinning medication, or known developmental delay. ^2^ ADHD (attention deficit hyperactivity disorder). ^3^ PUFA (polyunsaturated fatty acid). Horizontal line in table indicates that the intervention and assessment of adverse events was ongoing for the 12 week period.

**Table 3:** Children’s response to finger-prick blood collection.

	Sad Face	Neutral	Happy Face
Number of responses (n = 69)	14	8	47
% of total responses	20.3	11.6	68.1

**Table 4:** Parents/caregiver response to finger-prick blood collection.

	Completely Disagree	Somewhat Disagree	Not Sure	Somewhat Agree	Completely Agree
Number of responses (n = 70)	1	1	2	10	56
% of total responses	1.4	1.4	2.9	14.3	80.0

**Table 5:** Completion rate of outcome measures and compliance with tasks by children.

Outcome Measure	Completed Assessment n (%)	Of Completers, Those Who Also Complied n (%)	Completed Assessment n (%)	Of Completers, Those Who Also Complied n (%)
	Baseline		Post-Intervention	
HTKS	71/76 (93)	71/71 (100)	56/58 (97)	56/56 (100)
Mr Ant	74/76 (97)	74/74 (100)	57/58 (98)	57/57 (100)
Go/No-Go	74/76 (97)	72/74 (97)	56/58 (97)	56/56 (100)
EEG Go/No-Go	68/76 (89)	66/68 (97)	52/58 (90)	52/52 (100)
EEG Eyes-Open	69/76 (91)	67/69 (97)	52/58 (90)	52/52 (100)
EEG Eyes-Closed	66/76 (87)	66/66 (100)	52/58 (90)	52/52 (100)

## Data Availability

The data presented in this study are available on request from the corresponding author.
